# Utility of Radiomics for Predicting Patient Survival in Hepatocellular Carcinoma With Portal Vein Tumor Thrombosis Treated With Stereotactic Body Radiotherapy

**DOI:** 10.3389/fonc.2020.569435

**Published:** 2020-10-14

**Authors:** Kui Wu, Yongjie Shui, Wenzheng Sun, Sheng Lin, Haowen Pang

**Affiliations:** ^1^Department of Radiation Oncology, The Second Affiliated Hospital, Zhejiang University School of Medicine, Hangzhou, China; ^2^Department of Oncology, The Affiliated Hospital of Southwest Medical University, Luzhou, China

**Keywords:** hepatocellular carcinoma, portal vein tumor thrombosis, stereotactic body radiotherapy, radiomics, outcome prediction

## Abstract

**Introduction:** This study aimed to develop and validate the combination of radiomic features and clinical characteristics that can predict patient survival in hepatocellular carcinoma (HCC) with portal vein tumor thrombosis (PVTT) treated with stereotactic body radiotherapy (SBRT).

**Materials and Methods:** The prediction model was developed in a primary cohort of 70 patients with HCC and PVTT treated with SBRT, using data acquired between December 2015 and June 2017. The radiomic features were extracted from computed tomography (CT) scans. A least absolute shrinkage and selection operator regression model was used to build the model. Multivariate Cox-regression hazard models were created for analyzing survival outcomes and the radiomic features and clinical characteristics were presented with a nomogram. The area under the receiver operating characteristic curve (AUROC) was used to evaluate the model. Participants were divided into a high-risk group and a low-risk group based on the radiomic features.

**Results:** A total of four radiomic features and six clinical characteristics were extracted for survival analysis. A combination of the radiomic features and clinical characteristics resulted in better performance for the estimation of overall survival (OS) [area under the curve (AUC) = 0.859 (CI: 0.770–0.948)] than that with clinical characteristics alone [AUC = 0.761 (CI: 0.641–0.881)]. These patients were divided into high-risk and low-risk groups according to the radiomic features.

**Conclusion:** This study demonstrated that a nomogram of combined radiomic features and clinical characteristics can be conveniently used to assess individualized preoperative prediction of OS in patients with HCC with PVTT before SBRT.

## Introduction

Hepatocellular carcinoma (HCC) is the sixth most prevalent cancer worldwide, and the third leading cause of cancer-related deaths (1). China accounts for more than 50% of the global incidence of HCC and HCC is the fourth most commonly diagnosed cancer (2). Macrovascular invasion, where tumor cells invade the portal vein, hepatic veins, or the inferior vena cava in the liver (3, 4), is common in HCC. Portal vein tumor thrombosis (PVTT) is one of the most serious complications of HCC and has an incidence ranging from 44 to 62.2% (5). Between 10 and 60% of patients with HCC already have PVTT at the time of diagnosis (6, 7). This condition is strongly correlated with poor prognosis and the natural median survival time of patients with HCC and PVTT is only 2–4 months (8, 9).

Several clinical studies have confirmed that radiotherapy is effective for treating HCC with PVTT (10–12). Shui et al. (13) have shown stereotactic body radiotherapy (SBRT) can be used as the first-line therapy for HCC patients with extensive PVTT originally considered unsuitable for surgical resection or TACE. SBRT has emerged as a new radiotherapy technology that can deliver high doses of radiation to the target area in fewer fractions (14, 15). SBRT can accurately transfer a large dose of multiple beams to the target tumor within 1–5 fractions, owing to technical progress in accurate dose transfer, respiratory movement management, and daily image guidance. The relatively short treatment process can benefit patients by reducing interference with other treatment measures. Hence, we typically recommend SBRT to patients with unresectable HCC with PVTT undergoing multidisciplinary treatment. The purpose of SBRT is to reduce tumor thrombus and retain sufficient portal vein blood flow to allow the beneficial effect of any follow-up treatment.

Studies have also investigated the possibility of using radiomics as a potential prognostic indicator in oncology, specifically to classify patients and assess their risk categories, to develop personalized oncological treatments (16–18). The aim of this study was to develop a combination of radiomic features and clinical characteristics to estimate the overall survival (OS) in patients with HCC with PVTT treated using SBRT. Although numerous studies have been published on the use of radiomics in several cancer-outcome prediction models (19–21), and the correlation between the characteristics of radiation and the results of radiotherapy, few studies have focused on HCC with PVTT treated using SBRT. Therefore, our study aimed to develop and validate the combination of radiomic features and clinical characteristics that can predict patient survival in HCC with PVTT treated with SBRT.

## Materials and Methods

### Patient Selection

All patients (*n* = 70) who were treated at the Second Affiliated Hospital, Zhejiang University School of Medicine from December 2015 to June 2017 were included in the study. Treatment and data analysis were conducted according to the Declaration of Helsinki. Ethical approval for retrospective data analysis was obtained from the Second Affiliated Hospital, Zhejiang University School of Medicine Ethics Committee. The diagnosis of liver cancer was based on the guidelines of the American Association for the Study of Liver Diseases (22). Portal vein invasion was determined by the presence of filling defects in a low attenuation cavity near the primary tumor, as observed on enhanced computed tomography (CT).

In this study, patients received SBRT according to the following criteria: [1] tumor thrombus involving the main portal vein and/or the first portal vein, which was deemed unsuitable for surgery or transarterial chemoembolization; [2] Eastern Cooperative Oncology Group (ECOG) performance status (PS) score of 0–1; [3] absence of refractory ascites; [4] Child-Pugh class A, B, and C; [5] no previous history of radiotherapy for the liver; and [6] availability of more than 700 cm^3^ of unaffected liver.

### SBRT

The gross tumor volume (GTV) represents the extent of tumor thrombosis visualized on contrast-enhanced CT and magnetic resonance imaging (MRI). If the extent of primary liver disease was small (<5 cm) and adjacent to the PVTT, both were considered to be a part of the GTV. A total dose of 25–50 Gy was prescribed in five fractions over 5–7 days based on the GTV. SBRT plans were generated using the Varian radiation treatment planning system (Eclipse software, Varian Medical Systems, Palo Alto, CA, USA). Treatment was delivered with a Varian Trilogy linear accelerator (Varian Medical Systems, Palo Alto, CA) using a 6-MV photon beam.

### Follow-Up

The cutoff date for the last follow-up was February 28, 2018, for censored data analysis. The OS was calculated from the start of SBRT to the date of death or the last follow-up visit.

### Image Acquisition

The entire image used for radiomic analysis was obtained from the CT scan acquired prior to SBRT. Contrast-enhanced CT imaging was performed using a LightSpeed RT 16 scanner (GE Healthcare, Chicago, IL, USA). The scanning parameters used in this study were as follows: tube voltage, 120 kVp; field of view, 250–400 mm; pixel size, 512 × 512; slice thickness, 0.25 cm; and average number of slices, 116. The CT images were preprocessed by wavelet-based methods and then analyzed to extract the radiomic features from the GTV that contributed to the SBRT plans. Feature extraction was based on the three-dimensional (3D) slicer platform and performed using the pyradiomics package, which is available at: http://PyRadiomics.readthedocs.io/en/latest/ (accessed on June 30, 2019) (23).

### Statistical Analyses

All statistical analyses were performed using R software, version 3.6.3 (R Foundation for Statistical Computing, Vienna, Austria) and X-tile software, version 3.6.1 (Yale University School of Medicine, New Haven, Conn). Least absolute shrinkage and selection operator (LASSO) Cox regression modeling was used for data dimension reduction, feature selection, and radiomic feature building to select the most valuable predictive radiomic features from GTV. Multivariate Cox-regression hazard models were built for the survival outcome, radiomic features, and clinical characteristics presented with the nomogram. A nomogram is a specific functional representation that graphically displays prediction models using lines with numerical scales based on traditional statistical methods. LASSO was used to select radiomic features to fit the Cox proportion model using the “glmnet” package in R software, and the Multivariate Cox-regression hazards models and nomogram and calibration curve were performed with the “survival” and “rms” packages in R software, respectively. The area under the receiver operating characteristic (AUROC) curve was used to evaluate the nomogram model. The radiomic scores (Rad-scores) were calculated for each patient using a linear combination of selected radiomic features, weighted by their respective coefficients. The cutoff value of the Rad-score was calculated using X-tile software to categorize patients into the high-risk or low-risk groups.

## Results

**Figure 1:**
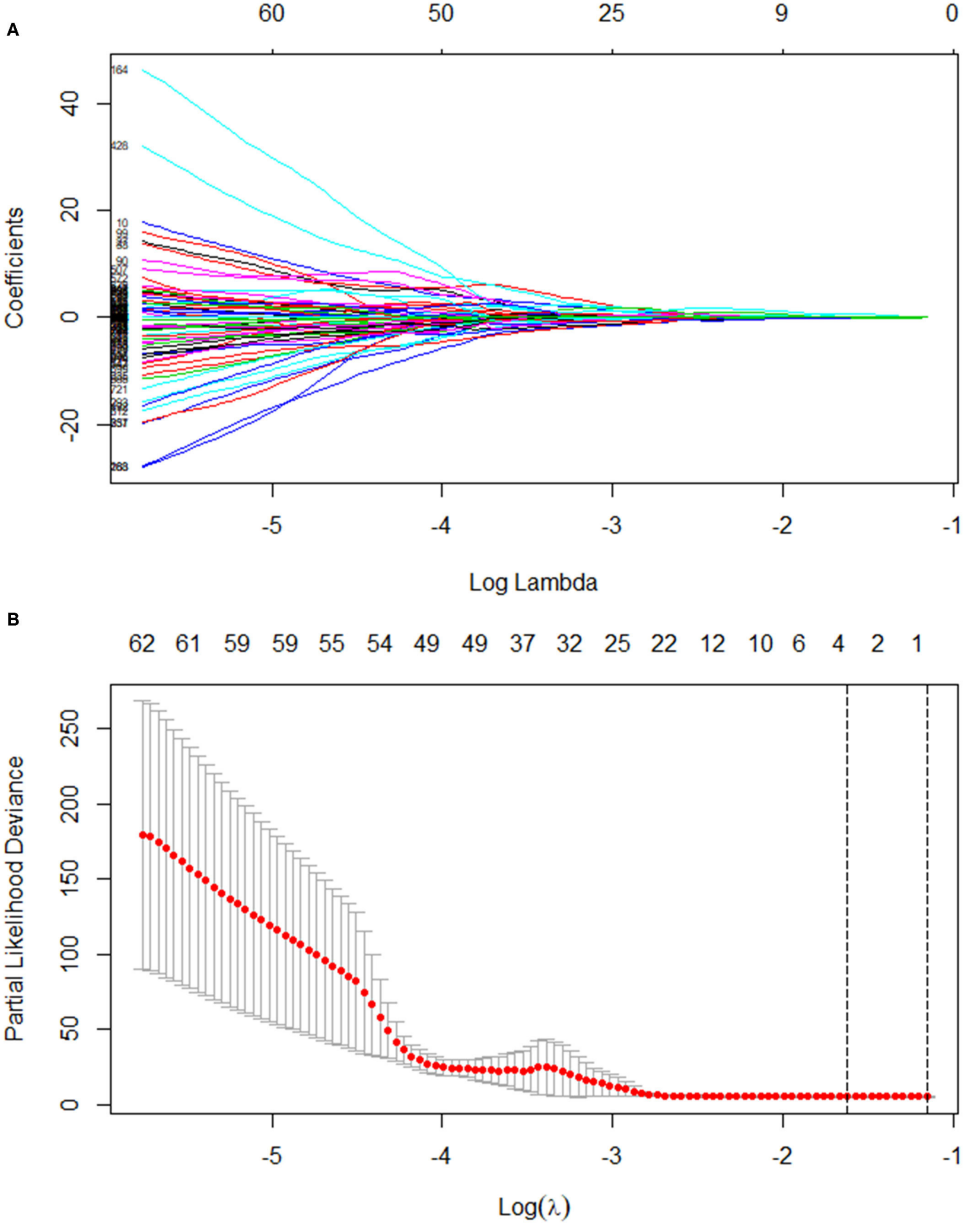
LASSO coefficient profiles of the 851 texture features **(A)** Tuning parameter (λ) selection in the LASSO model used 10-fold cross-validation with minimum criteria **(B)**. LASSO, least absolute shrinkage and selection operator.

The median follow-up time was 9.5 months. Twenty-five patients (35.7%) were still alive at the time of the current analysis. The median survival time was 10.0 months (95% CI, 7.7–12.3). Table 1 shows the patients' clinical characteristics. All 851 radiomic features were extracted, including Shape features (which include descriptors of the 2D and 3D size and shape of the region of interest and are independent from the gray level intensity distribution in the region of interest and therefore only calculated on the non-derived image and mask), First Order features (which describe the distribution of voxel intensities within the image region defined by the mask through commonly used and basic metrics), Gray Level Co-occurrence Matrix features (GLCM, which describe the second-order joint probability function of an image region constrained by the mask), Gray Level Dependence Matrix features (GLDM, which quantify gray level dependencies in an image), Gray Level Run Length Matrix features (GLRLM, which quantify gray level runs that are defined as the length in number of consecutive pixels that have the same gray level value), Gray Level Size Zone Matrix features (GLSZM, which quantify gray level zones in an image), and Neighboring Gray Tone Difference Matrix features (NGTDM, which quantify the difference between a gray value and the average gray value of its neighbors within some distance). High-throughput radiomic features were reduced with LASSO regression (Figure 1). Four radiomic features and six clinical characteristics were extracted for OS analysis. The radiomic features included Short Run Low Gray Level Emphasis (SRLGLE, which measures the joint distribution of shorter run lengths with lower gray-level values) of the GLRLM of the wavelet-HLL (H = high-frequency band, L = low-frequency band) (feature 1), Inverse Difference Moment Normalized (Idmn, which is a measure of the local homogeneity of an image) of the GLCM of the wavelet-LLL (feature 2), Small Dependence Low Gray Level Emphasis (SDLGLE, which measures the joint distribution of small dependence with lower gray-level values) of the GLDM of the wavelet-HLL (feature 3), and Idmn of the GLCM of the original (feature 4). The clinical characteristics included the ECOG score, type of PVTT, Child-Pugh classification, age, and albumin and hemoglobin levels. Table 2 summarizes the results of the univariate log-rank test for clinical characteristics.

**Table 1:** Patient characteristics.

**Characteristics**	***n* (%)**
**Age, y**	
≥50	48 (68.6)
<50	22 (31.4)
**Gender**	
Male	59 (84.3)
Female	11 (15.7)
**Stage T**	
T3	65(92.9)
T4	5(7.1)
**Stage N**	
N0	48(68.6)
N1	22(31.4)
**Stage M**	
M0	57(81.4)
M1	13(18.6)
**Types of PVTT**	
II	42 (60.0)
III	27 (38.6)
IV	1 (1.4)
**HBsAg**	
Negative	12 (17.1)
Positive	58 (82.9)
**Child-Pugh classification**	
A	45 (64.3)
B	24 (34.3)
C	1 (1.4)
**ECOG**	
0	56 (80.0)
1	14 (20.0)
**AFP, ng/L**	
≤20	13 (18.6)
21~399	17 (24.3)
≥400	40 (57.1)
**PLT, 10**^**9**^**/L**	
≥100	39 (55.7)
<100	31 (44.3)
**HGB, g/L**	
≥120	42 (60.0)
<120	28 (40.0)
**TBIL**, **μmol/L**	
≥20	34 (48.6)
<20	36 (51.4)
**ALB, g/L**	
≥35	41 (58.6)
<35	29 (41.4)
**ALT, U/L**	
≥50	25 (35.7)
<50	45 (64.3)
**AST, U/L**	
≥50	48 (68.6)
<50	22 (31.4)

*PVTT, Portal vein tumor thrombus; HBsAg, Hepatitis B surface antigen; PS, Performance status; ECOG, Eastern Cooperative Oncology Group; AFP, Alpha–fetoprotein; PLT, Platelet; HGB, Hemoglobin; TBIL, Total bilirubin; ALB, Albumin; ALT, Alanine aminotransferase; AST, Aspartate aminotransferase*.

**Table 2:** Significant covariates with respect to the survival and related log-rank test *P*-values.

**Covariate**	**HR (95% CI for HR)**	***P*-value**
Age	1.04 (1.011–1.07)	0.006796
Gender	1.728 (0.8304–3.598)	0.1435
Stage T	0.7244 (0.2225–2.358)	0.5924
Stage N	0.943 (0.5012–1.773)	0.8546
Stage M	0.936 (0.435–2.014)	0.8656
Types of PVTT	0.518 (0.276–0.971)	0.0403
HBsAg	0.989 (0.9302–1.052)	0.7339
Child-Pugh classification	1.914 (1.036–3.537)	0.0243
ECOG	2.342 (1.232–4.453)	0.009441
AFP	1 (1)	0.06224
PLT	1 (0.9963–1.005)	0.8193
HGB	0.9783 (0.9628–0.994)	0.006954
TBIL	1.007 (0.9957–1.018)	0.2332
ALB	0.918 (0.8543–0.9865)	0.01982
ALT	0.997 (0.993–1.002)	0.2326
AST	0.999 (0.9981–1.001)	0.5197

*PVTT, Portal vein tumor thrombus; HBsAg, Hepatitis B surface antigen; PS, Performance status; ECOG, Eastern Cooperative Oncology Group; AFP, Alpha–fetoprotein; PLT, Platelet; HGB, Hemoglobin; TBIL, Total bilirubin; ALB, Albumin; ALT, Alanine aminotransferase; AST, Aspartate aminotransferase*.

**Figure 2:**
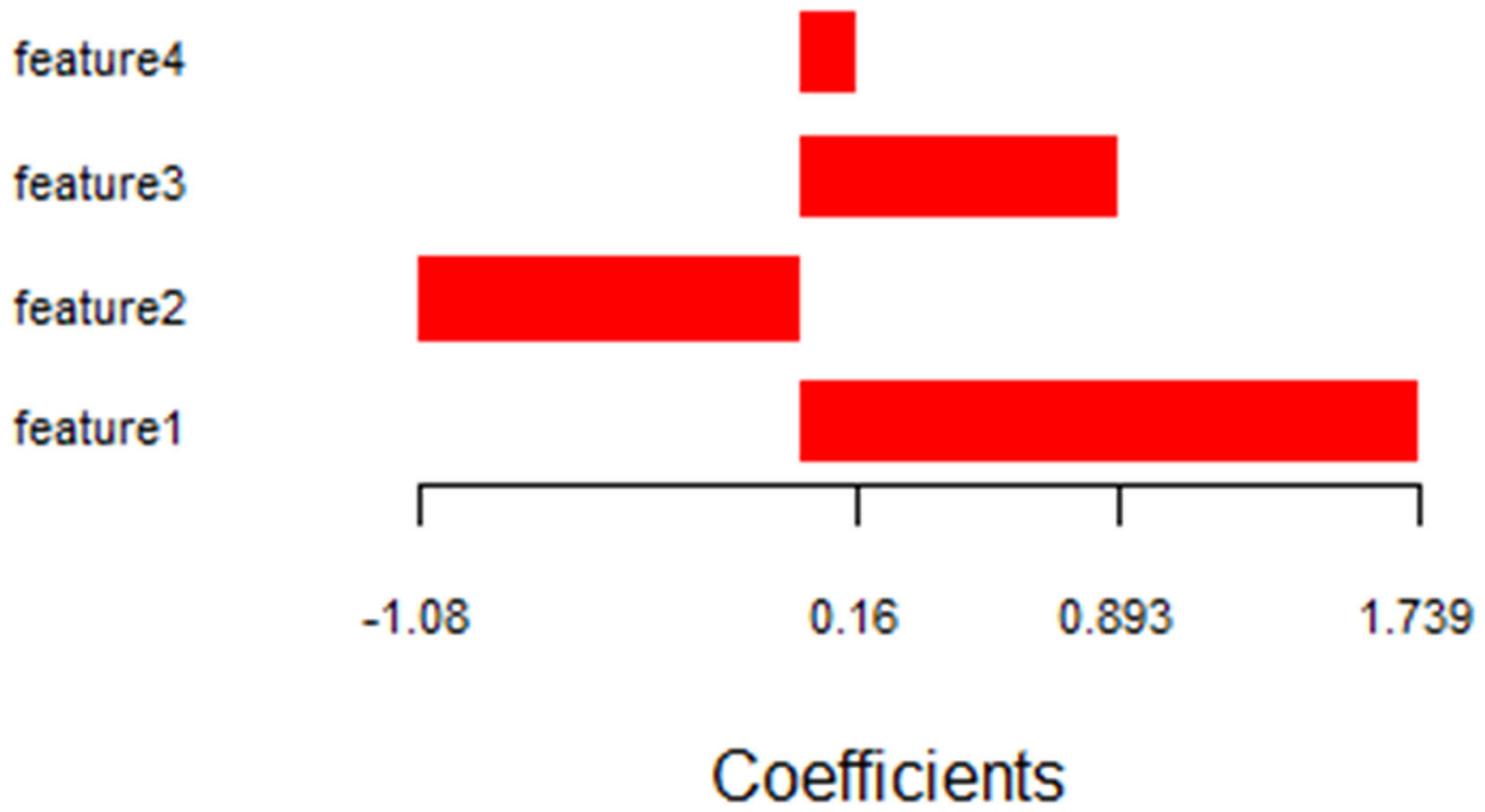
Histograms showing the role of individual features contributing to the radiomic features. Contributing features are plotted on the y-axis and their LASSO analysis coefficients are plotted on the x-axis. LASSO, least absolute shrinkage and selection operator.

The coefficients of the selected radiomic features are shown in Figure 2. Features 1–4 consisted of radiomic features and the Rad_score was calculated using the following formula:

$$\begin{array}{lll} \text{Rad\_score} & = & \text{feature }1\;\times \;1.7386385\;-\text{ feature }2\;\times \;1.0795126\\ + & \text{feature }3\;\times \;0.8927949\;+\text{ feature }4\;\times \;0.1599488 \end{array}$$

**Figure 3:**
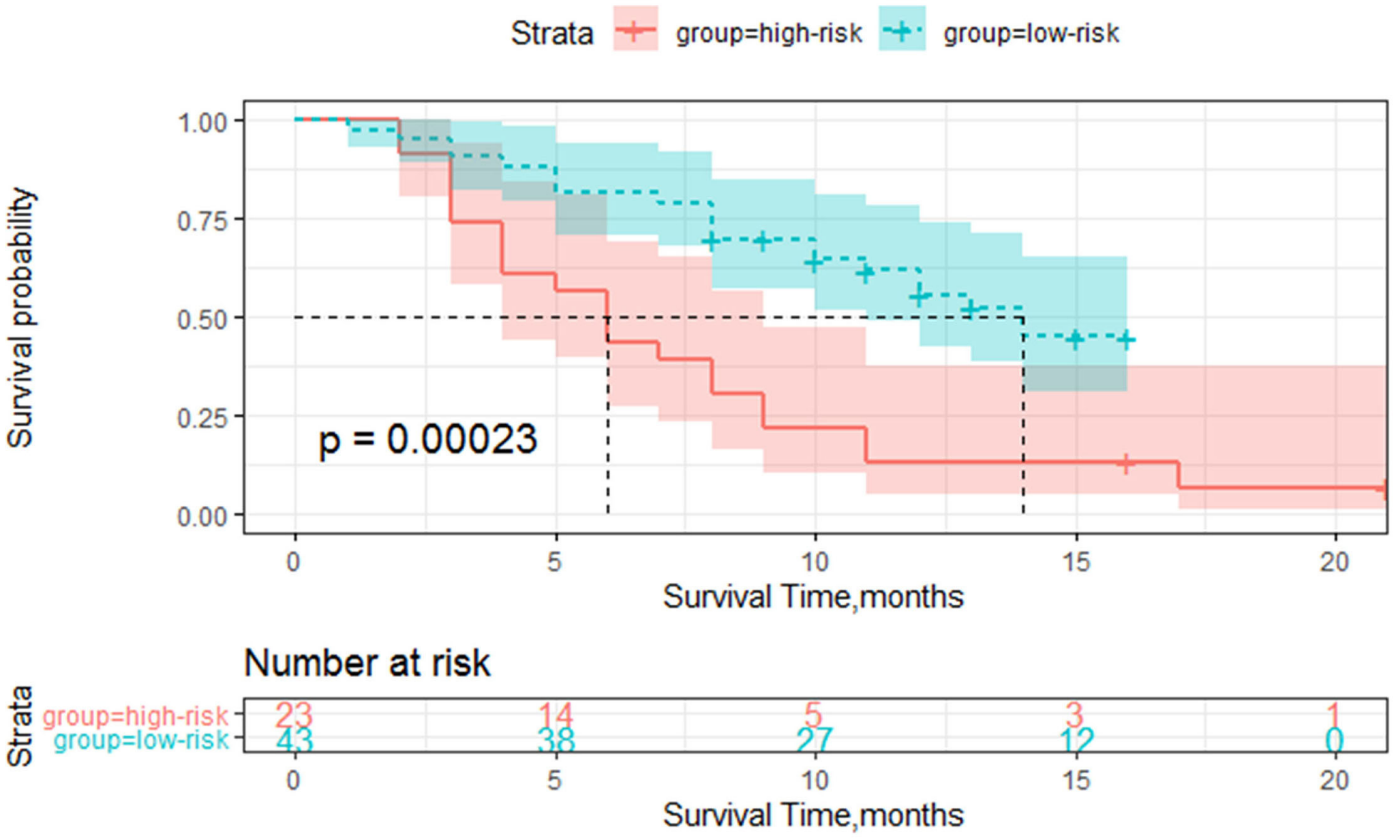
Survival curve of the high and low-risk groups based on the radiomics score classification.

The cutoff Rad-score value was −0.1, which was used to classify patients into the high-risk group (Rad-score ≥-0.1) and low-risk group (Rad-score < −0.1). The survival curves of both groups are shown in Figure 3.

**Figure 4:**
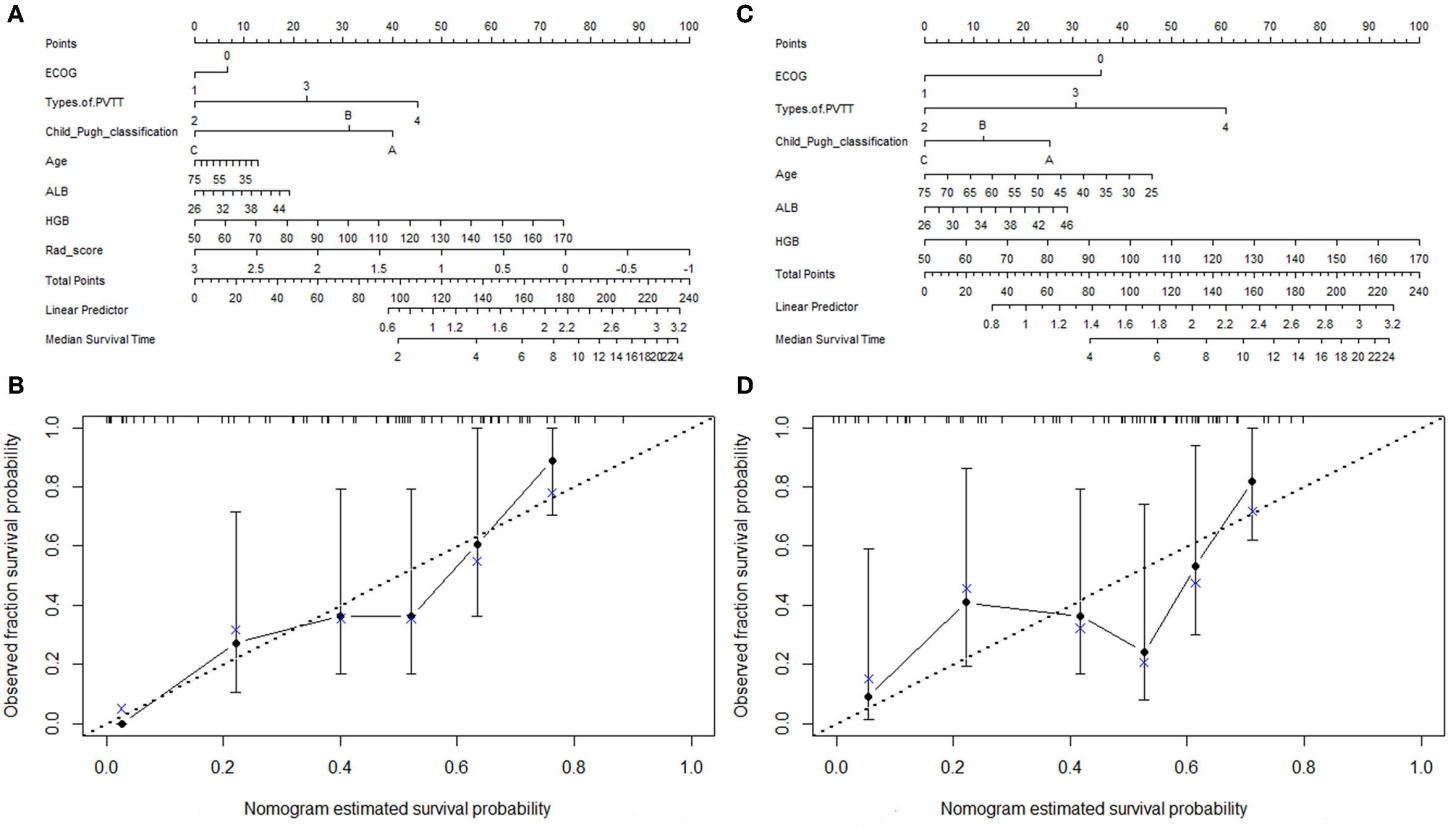
The nomogram of the combination of the radiomic features and clinical characteristics **(A)** and the associated calibration curve for the combination of the radiomic features and clinical characteristics. **(B)** The nomogram of the clinical characteristics **(C)** and the associated calibration curve for clinical characteristics **(D)**.

**Figure 5:**
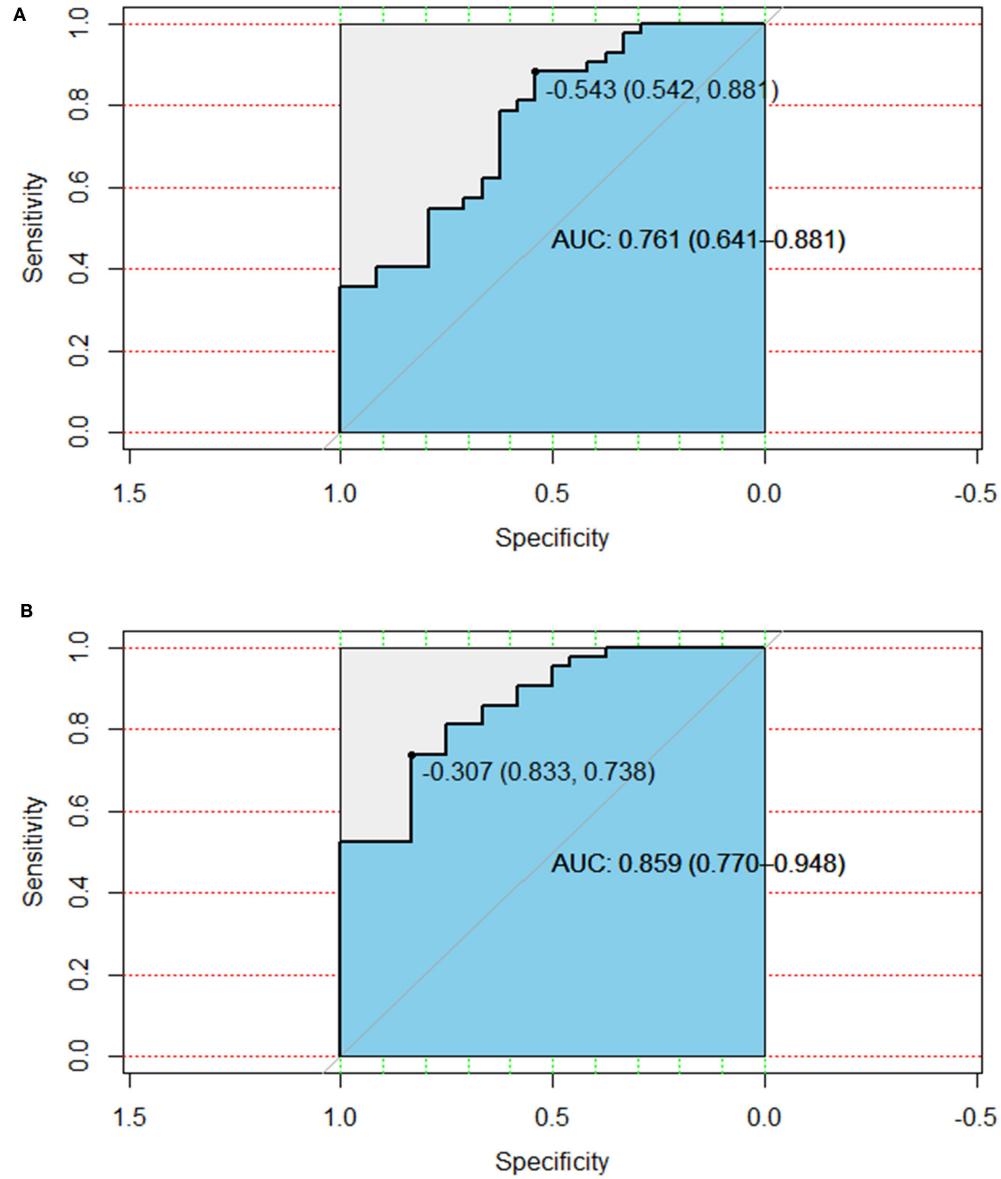
The AUC of clinical characteristics **(A)** and the combined radiomic features and clinical characteristics **(B)**. AUC, area under the curve.

The combination of the radiomic features, clinical characteristics nomogram, and calibration curves is presented in Figure 4. The area under the curve (AUC) for the clinical characteristics was 0.761 (CI: 0.641–0.881), and the AUC was 0.859 (CI: 0.770–0.948) when the radiomic features were combined with the clinical characteristics (Figure 5). We also compared our findings with those of recent studies (Table 3) (24–27).

**Table 3:** Summary of the studies that evaluated radiomics in the hepatocellular carcinoma.

**References**	**Purposes**	**Type**	**Treatment**	**Imaging modality**	**Feature selection model**	**Main results**
Ji et al. (24)	RFS	Early Stage HCC	Hepatectomy	Contrast-enhanced CT	LASSO	AUC 0.82
Shan et al. (25)	Predict early recurrence	HCC	Hepatectomy	Contrast-enhanced CT	LASSO	AUC 0.80
Zheng et al. (26)	Predict recur-rence and survival	Solitary HCC	Hepatectomy	Contrast-enhanced CT	LASSO	Recurrence AUC 0.64 Survival AUC 0.71
Peng et al. (27)	Prediction of MVI	HBV-related HCC	–	Contrast-enhanced CT	LASSO	c-index 0.846
In this study	Predict survival	HCC with PVTT	SBRT	Contrast-enhanced CT	LASSO	Survival AUC 0.859

*RFS, recurrence-free survival; HCC, hepatocellular carcinoma; LASSO, least absolute shrinkage and selection operator; MVI, microvascular invasion; HBV, hepatitis B virus; SBRT, stereotactic body radiotherapy*.

## Discussion

The application of radiomics has been extensively studied in esophageal cancer (28, 29), non-small cell lung cancer (30), breast cancer (31), nasopharyngeal carcinoma (32), Glioblastoma (33), and rectal cancer (34), which indicates the potential of radiomics for predicting the efficacy of treatment or patient prognosis. Radiotherapy-orientated CT imaging must be acquired prior to SBRT treatment of HCC with PVTT. Image data analysis of the pre-SBRT CT image is used to predict the OS of patients with HCC and PVTT, to limit examinations and provide guidance for clinical treatment decisions. This knowledge provided the basis for this retrospective study. We developed and validated a nomogram based on a combination of radiomic features and clinical characteristics from localized CT performed prior to SBRT treatment to make individualized OS predictions in patients with HCC with PVTT. The nomogram included four radiological features and six clinical features. The methodology implemented in this study is simple and reproducible because the features were generated from a validated package, which is freely available from the 3D slicer (23).

LASSO regression is suitable for the accurate analysis of large radiological features with relatively small sample sizes and its design can prevent overfitting of the model (35, 36). The regression coefficients of most features are reduced to zero during the model fitting process, making it easier to interpret the model, which allows for the identification of features closely related to OS. Yin et al. (37) compared three feature selection methods (relief, LASSO, and random forest), and concluded that LASSO had the best performance, which could enhance the application of radiomics methods. The radiological features identified successfully classified patients into high-risk and low-risk groups, based on the Rad-scores.

SBRT focusses on treating stage IIIA and IVB HCC with PVTT, which has a relatively short OS. The accurate prediction of the OS of patients with HCC with PVTT undergoing SBRT will typically benefit those with shorter OS periods the most. We aim to develop a new model in a future study, which will include patients with low-stage HCC treated with SBRT. TNM staging was not selected as a clinical feature related to OS in this study because patients with HCC with PVTT belong to the late clinical-stage, which makes it difficult to predict OS using clinical staging since all patients have similar staging information. Alpha-fetoprotein was also not selected as a clinical feature to determine OS in this study for the same reason. This probably contributed to the poor predictive value of the clinical parameters in this study. The combination of the radiomic features and clinical characteristics resulted in better performance for the estimation of OS [AUC = 0.859 (CI: 0.770–0.948)] than that with the clinical characteristics alone [AUC = 0.761 (CI: 0.641–0.881)]. The radiomic features effectively compensated for the deficiencies in the clinical characteristics. This model also supported the value of radiomic features for the individual association between the OS of HCC with PVTT treated by SBRT.

The limitations of the study are that genomic characteristics were not considered. In recent years, genetic markers have been used to predict OS in patients with liver cancer in research settings (38). Radiogenomics is a discipline that studies the relationship between image phenotypes and genomics. It has gradually emerged in the field of cancer research and continues to receive more attention (39–41). Further research is necessary with a larger study population to identify the associated genetic characteristics and predict the OS of patients more accurately. Another limitation of this feasibility study is the lack of validation based on independent data sets. For the training sample size, Chalkidou et al. (42) proposed that for multiple regressions, at least 10–15 observations per predictor variable is required to produce reasonably stable estimates. In our study, four features were selected for the final model and the minimum data size was 40–60. Finally, 70 patients were involved in this study as training group, which were enough. Due to the limited sample size, we were unable to divide the survey cohort into testing groups. A separate multi-center validation study is currently underway and will enroll a larger patient population to overcome this limitation.

## Conclusions

This study demonstrated that the use of a nomogram combining radiologic features with clinical risk factors can personalize OS prediction in patients with HCC with PVTT who underwent SBRT.

## Data Availability Statement

All datasets generated in this study are included in the article/Supplementary Material.

## Ethics Statement

The studies involving human participants were reviewed and approved by treatment and data analysis were conducted according to the Declaration of Helsinki. The studies involving human participants were reviewed and approved by the Second Affiliated Hospital, Zhejiang University School of Medicine Ethics Committee. The patients/participants provided their written informed consent to participate in this study.

## Author Contributions

HP: guarantor of integrity of entire study. KW and HP: literature research. KW, YS, and HP: statistical analysis and manuscript editing. All authors: study concepts, study design, data acquisition, data analysis and interpretation, manuscript drafting and manuscript revision for important intellectual content, approval of final version of submitted manuscript, agree to ensure any questions related to the work are appropriately resolved, and clinical studies.

## Conflict of Interest

The authors declare that the research was conducted in the absence of any commercial or financial relationships that could be construed as a potential conflict of interest.
